# P-63. Direct Identification of Gram-Negative Bacteria Culture: Analytical and Clinical Performance of a Multi-pathogen Molecular Assay

**DOI:** 10.1093/ofid/ofaf695.292

**Published:** 2026-01-11

**Authors:** Seema Shaikh, Brian Bernier, Megan Martinez, Jason Williams, Nicole Kroutter, Mahima Pancholi, Edward Sekinger, Sundaresh Brahmasandra, Janet Farhang

**Affiliations:** Diasorin, Austin, TX; Diasorin, Austin, TX; Diasorin, Austin, TX; Diasorin, Austin, TX; Diasorin, Austin, TX; Diasorin, Austin, TX; Diasorin, Austin, TX; Diasorin, Austin, TX; Diasorin, Austin, TX

## Abstract

**Background:**

Bloodstream infection (BSI) is a critical condition requiring prompt diagnosis and treatment. This study evaluated the LIAISON PLEX^®^ Gram-Negative Blood Culture Assay, a multi-target molecular panel for rapid and accurate identification of gram-negative (GN) bacteria grown in blood culture specimens. The panel’s analytical and clinical performance was compared to standard culture and automated methods to assess its effectiveness as a faster, sensitive adjunct.

**Methods:**

The assay was performed directly on blood culture specimens positive for gram-negative bacteria. A 300 µL aliquot was processed in a single-use cartridge, utilizing NanoGrid Technology for rapid and direct detection without the need for target amplification. The fully automated LIAISON PLEX^®^ system extracts nucleic acid and hybridizes it to a microarray with probes for 19 bacteria and 8 resistance genes, using gold nanoparticle chemistry with silver-enhanced signal amplification.

**Results:**

The assay showed 100% positivity for GN bacteria and 0% for negative blood cultures in a growth and detection study. It detected up to 21 representative strains across 13 blood culture media types and showed 100% inclusivity to 245 of 246 on-panel strains, including resistance markers (RM). The assay maintained sensitivity with low concentrations of on-panel GN organisms in the presence of high levels of other co-infecting GN or gram-positive bacteria.

Clinical performance was assessed in a multi-site study with 381 prospective, 231 pre-selected, and 746 contrived specimens. Among prospective samples, Positive Percent Agreement (PPA) ranged from 96.6% (*Pseudomonas aeruginosa*) to 100% (10 organisms including RM) excluding *Acinetobacter* spp., where the PPA was lower due to limited prevalence, and Negative Percent Agreement (NPA) ranged from 98.8% (*E. coli*) to 100% (20 organisms including RM). The common pathogens identified were *Escherichia coli* (47.2%) and *Klebsiella pneumoniae* (12.8%). In contrived specimens, PPA ranged from 97.2% to 100%, and NPA from 99.6% to 100%.

**Conclusion:**

The LIAISON PLEX^®^ Gram-Negative Blood Culture Assay offers fast and accurate identification of gram-negative pathogens, providing a reliable adjunct to culture-based methods for timely BSI management.

**Disclosures:**

Seema Shaikh, MS, Diasorin: Employee Brian Bernier, PhD, Diasorin: Employee Megan Martinez, BS, Diasorin: Employee Jason Williams, BS, Diasorin: Employee Nicole Kroutter, MS, Diasorin: Employee Mahima Pancholi, MS, Diasorin: Employee Edward Sekinger, PhD, Diasorin: Employee Sundaresh Brahmasandra, PhD, Diasorin: Employee Janet Farhang, PhD, Diasorin: Employee|Diasorin: Employee

